# Improved Biosafety and Biosecurity Measures and/or Strategies to Tackle Laboratory-Acquired Infections and Related Risks

**DOI:** 10.3390/ijerph15122697

**Published:** 2018-11-29

**Authors:** Huasong Peng, Muhammad Bilal, Hafiz M. N. Iqbal

**Affiliations:** 1 State Key Laboratory of Microbial Metabolism, School of Life Sciences and Biotechnology, Shanghai Jiao Tong University, 800 Dongchuan Road, Shanghai 200240, China; 2 School of Life Science and Food Engineering, Huaiyin Institute of Technology, Huaian 223003, China; bilaluaf@hotmail.com; 3 Tecnologico de Monterrey, School of Engineering and Sciences, Campus Monterrey, Ave. Eugenio Garza Sada 2501, CP 64849 Monterrey, N.L., Mexico; hafiz.iqbal@itesm.mx

**Keywords:** laboratory-acquired infections, biological risks, biosafety and biosecurity measures, biohazards, life-threatening diseases

## Abstract

Herein, we reviewed laboratory-acquired infections (LAIs) along with their health-related biological risks to provide an evidence base to tackle biosafety/biosecurity and biocontainment issues. Over the past years, a broad spectrum of pathogenic agents, such as bacteria, fungi, viruses, parasites, or genetically modified organisms, have been described and gained a substantial concern due to their profound biological as well as ecological risks. Furthermore, the emergence and/or re-emergence of life-threatening diseases are of supreme concern and come under the biosafety and biosecurity agenda to circumvent LAIs. Though the precise infection risk after an exposure remains uncertain, LAIs inspections revealed that *Brucella* spp., *Mycobacterium tuberculosis*, *Salmonella* spp., *Shigella* spp., *Rickettsia* spp., and *Neisseria meningitidis* are the leading causes. Similarly, the human immunodeficiency virus (HIV) as well as hepatitis B (HBV) and C viruses (HCV), and the dimorphic fungi are accountable for the utmost number of viral and fungal-associated LAIs. In this context, clinical laboratories at large and microbiology, mycology, bacteriology, and virology-oriented laboratories, in particular, necessitate appropriate biosafety and/or biosecurity measures to ensure the safety of laboratory workers and working environment, which are likely to have direct or indirect contact/exposure to hazardous materials or organisms. Laboratory staff education and training are indispensable to gain an adequate awareness to handle biologically hazardous materials as per internationally recognized strategies. In addition, workshops should be organized among laboratory workers to let them know the epidemiology, pathogenicity, and human susceptibility of LAIs. In this way, several health-related threats that result from the biologically hazardous materials can be abridged or minimized and controlled by the correct implementation of nationally and internationally certified protocols that include proper microbiological practices, containment devices/apparatus, satisfactory facilities or resources, protective barriers, and specialized education and training of laboratory staffs. The present work highlights this serious issue of LAIs and associated risks with suitable examples. Potential preventive strategies to tackle an array of causative agents are also discussed. In this respect, the researchers and scientific community may benefit from the lessons learned in the past to anticipate future problems.

## 1. Introduction

**Figure 1:**
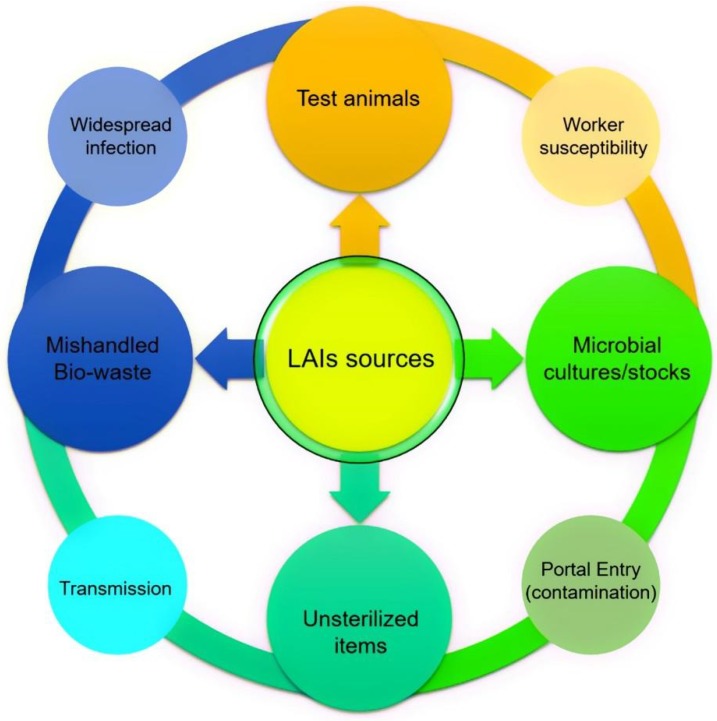
Laboratory-acquired infections (LAIs) sources, the outer circle shows a possible chain of infection causing route.

The ever-increasing scientific advancements and growing interests in microbial orientated bioresearch at different laboratory levels can have both positive and negative impacts. Microbially-orientated LAIs are serious biohazards not only for the environment but, also, for public health, in particular, laboratory workers, who are exposed through various routes. LAIs sources along with a possible chain of infection causing route are shown in Figure 1. Several factors play a crucial role in LAIs exposure and transmission. For instance, inhalation of contagious aerosols, contact with mucous membranes by splash, touch, or spill, or infection via the percutaneous route, i.e., bites, cuts, accidental self-inoculation, etc. are potential LAIs routes. Nevertheless, in most of the LAI cases, the underlying possible LAIs routes remain poorly defined [1,2]. Despite that, LAIs and/or LAIs-related biohazards can be abridged or controlled by employing internationally recognized protocols and hazards-free laboratory procedures [3]. For instance, by maintaining the following principles: (1) primary and secondary barriers, (2) personal and procedural barriers, (3) protective barriers, (4) proper microbiological techniques, (5) proper culture handling procedures, (6) proper (bio)-waste management, (7) adequate facilities such as appropriate sterilization or decontamination services, (8) adequate protection and deprotection steps, and (9) up to date training and first aid awareness to laboratory workers, etc. Considering the above points, working with infectious microbial strains such as *Brucella* spp., *Mycobacterium tuberculosis*, *Salmonella* spp., *Shigella* spp., *Rickettsia* spp., and *Neisseria meningitidis* among others, necessitates the use of suitable laboratory practices along with properly defined precautionary measures to ensure the safety of laboratory workers from LAIs [4]. Based on literature evidence, the first LAIs diseases were reported at the time of Pasteur and Koch, and these have been reported since 1897 [5].

**Figure 2:**
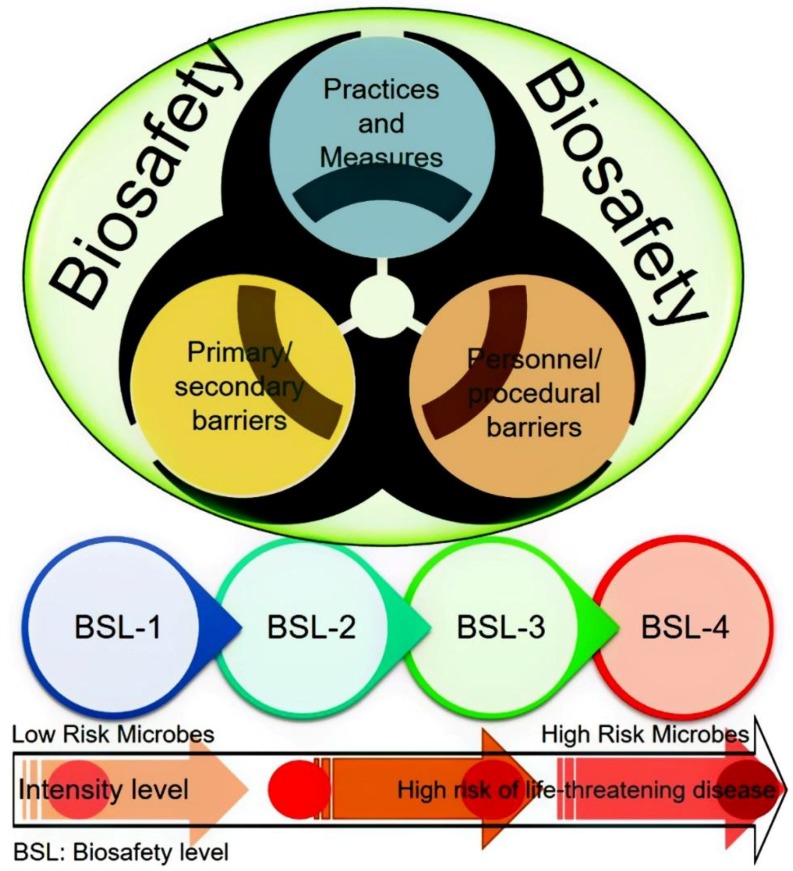
A schematic representation of a biosafety concept and four biosafety levels (BSL) with their risk intensity.

There has been increasing apprehension among the general public and scientific community, around the globe, regarding the potential for bioterrorism and accidental laboratory escape of potential pandemic pathogenic microorganisms [6]. This serious concern has forced the debate regarding the controlled access to high-consequence pathogens and toxins, and improved biosafety measures, particularly for pathogens capable of disseminating rapidly in the environment [7,8,9]. In the settings with well-established regulatory environments accompanied by the robust implementation, the risk of pathogen escape was found to be low according to many experts. Nonetheless, to achieve this goal, it is prerequisite for research and diagnostic laboratories to enforce and implement strict biosafety/biosecurity practices and to possess well-trained personnel, especially those with biosafety level (BSL) 3 or higher containment level facilities [10]. Figure 2 illustrates a schematic representation of a biosafety concept and four biosafety levels with their risk intensity.

Laboratory biosafety and/or biosecurity are broader terms that refer to a set of precautionary measures for safe handling of pathogenic microbial strains and hazardous biological waste materials. The above-mentioned terms, i.e., (1) biosafety, and (2) biosecurity are often denoted with similar meanings in the scientific literature. However, the distinctions between these two concepts have been indicated academically. Bakanidze et al. [11] specified that the biosafety and/or biosecurity concepts comprise on numerous strategic measures that may overlap each other but the aim stays the same to control LAIs. In order to give clear insight and to avoid misinterpretation, biosafety involves all the preventive measures undertaken to eliminate pathogenic microorganisms and their potential toxins [6]. On the other hand, biosecurity encompasses a set of preventive strategies designed to reduce the menace of transmission of infectious diseases in crops and livestock, isolated pests, or genetically modified organisms (GMOs) [12]. Herein, an effort has been made to highlight this serious issue of LAIs. The focus has also been given to highlight associated risks with suitable examples. The information is also given on potential preventive strategies to tackle an array of causative agents such as bacteria, fungi, viruses, parasites, or genetically modified organisms. Towards the end, lessons learned, and future perspectives and recommendations are given to circumvent LAIs.

## 2. LAIs and Associated Risks

The term LAIs refer to all infections acquired through laboratory work or laboratory-associated activities with or without the onset of infections and generally consequences from occupational acquaintance to infectious agents [13]. Literature survey revealed only a scarce report of LAIs and accidents by the use of genetically modified organisms (GMOs) [1]. These infections can occur during investigational or research work in biological settings such as microbiological (bacteria, fungi, virus) or animal facilities. Though LAIs and epidemics related to risk group 4 are particularly very rare, the establishment of biosafety level-4 (BSL-4) laboratories setup is indispensable to scrutinize and investigate new emerging diseases with considerable threats [14]. Also, proper education and training are essential for identifying and circumventing the outbreak. Worthy to mention is that the LAIs pose a significant issue of concern to the public/community health since an infected laboratory employee may prove to be a transmission risk for other people. Nevertheless, the scientific reports regarding LAIs are quite limited [15], and primarily based on internal data of the infection laboratory or by the official investigation. On the contrary, numerous reports about LAIs in traditional research laboratories have recently been documented in the scientific literature [16]. Siengsanan-Lamont and Blacksell [17], recently reviewed that a total number of 27 LAIs reports were published between 1982 and 2016 in Asia-Pacific (Table 1) [18]. Among these reports, 56%, 26%, 11%, and 2% were reported in East Asia, Oceania, Southeast Asia, and South Asia, respectively. A total of 78% of those LAIs originated from developed countries, including Australia, Japan, South Korea, Taiwan, and Singapore, and 19% of reports were stated in China, India, and Malaysia. Inhalation, in particular by aerosols, percutaneous inoculation (for example, needlestick injuries, broken glass injury, or animal bites), direct contact to adulterated/infected surfaces (gloves, hands), or ingestion (eating, smoking, or accidental aspiration) are considered to be the most prevalent routes of infection [15]. Therefore, the laboratory staff necessarily ponder the transmission route and infective dose for humans, which differ according to the inoculation route [19]. The accelerated disease risk for microbiology-related laboratory workers working on zoonotic agents has long been recognized. Undoubtedly, LAIs caused by exposure to pathogenic bacterial microorganisms has been reported as the most common, and viruses-related LAIs have also escalated in recent years [20].

## 3. Studies of Laboratory-Acquired Infections (LAIs)

LAIs that have occurred due to an array of bacteria, viruses, fungi, rickettsiae, and parasites have been reported in the scientific literature. In 1976, Pike [21] carried out the largest survey of LAIs, reporting that 4079 of them were due to 159 agents, although ten agents accounted for 150% of the cases [21,22]. At least 173 deaths were caused by LAIs [23]. A survey at UK laboratories from 1994–1995 reported the predominance of gastrointestinal infections, in particular, of shigellosis [24]. Comparable data were, also, recorded from a survey of clinical microbiology laboratories in Utah from 1978 to 1992, with shigellosis being the leading cause of LAIs [25]. These reports advocate a shift in the pattern of LAIs, with enteric infections prevailing. Nevertheless, no denominator data was provided to conclude the actual incidence or risk of infection for laboratory staff/workers. In a 2002–2004 inspection of clinical laboratory directors, who participated in ClinMicroNet, an online forum sponsored by the American Society of Microbiology, approximately 33% of laboratories stated the incidence of at least one LAI [26]. Among these infections, brucellosis, shigellosis, and salmonellosis were found to be the most common LAIs. Notably, the annual number of LAIs has gradually reduced since 1965 [23]. For instance, survey results from the UK during the period 1988–1989 recorded an infection occurrence of 82.7 cases per 100,000 person-years, in contrast with a frequency of 16.2 cases per 100,000 person-years during the period 1994–1995 [24,27]. These findings undeniably indicate a greater awareness of the threats of working with infectious agents and the implementation of higher laboratory safety.

## 4. Specific Laboratory-Acquired Infections and Prevention

### 4.1. Laboratory-Acquired Brucellosis and Prevention Strategies

Brucellosis has been recognized as one of the most significant causes of LAIs [15,28]. Reports have shown that many of the infections are acquired through workers being unaware of contaminated/polluted microbial cultures from clinical cases [29,30]. From 1979 to 2015, brucellosis has been reported as triggering 378 LAIs [31], and in 80% of the *Brucella*-associated LAIs, *Brucella melitensis* was found to be the major causative agent [15]. In their study, Traxler group [15] revealed that amongst the 167 potential *Brucella*-exposed employees, 71 developed LAI brucellosis. Improper use of the biological safety cabinets, as well as, lack of *Brucella* spp. (belonging to risk group Level 3) recognition isolated by laboratory staff are the important causes of Brucellosis outbreaks [30]. On the other hand, the onset of laboratory-acquired brucellosis is not always related to the occupational accident but can occur due to direct contact, contaminated skin, needle stick injuries, and splashing in the conjunctivae, or mucous membranes [32]. There are some reported cases of brucellosis infections that occurred following eating or drinking near a culture-processing workbench, and improper individual preventive measures while dealing with the contagious material [32]. Lack of proper awareness for *Brucella* spp. pathogenicity and insufficient related to handling biohazard materials could also be a causative source to new infections [33].

### 4.2. Laboratory-Acquired Tuberculosis and Prevention Strategies

**Figure 3:**
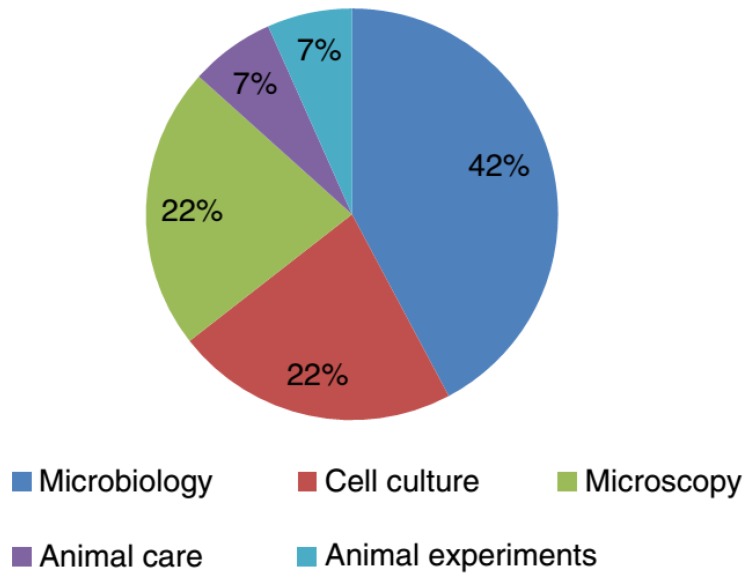
In which context did the infection happen? Reproduced from Wurtz et al. [36], with permission from Springer Nature. Copyright © 2016, Springer-Verlag Berlin Heidelberg.

Initial inspections of laboratory-acquired tuberculosis documented the prevalence of pathogenic *Mycobacterium tuberculosis* three to nine times higher amongst laboratory employees compared with the general population [34]. Nonetheless, laboratory-associated tuberculosis is extremely challenging to recognize owing to the wide-environmental dissemination of these microorganisms and chronicity of the infection [35]. The extreme menace of LAI for laboratory staffs handling *M. tuberculosis* is related to the aerosols generation. Also, the literature survey revealed some *M. tuberculosis* cases occurred due to inadequate isolation techniques and high capacities of specimens handled. It is important to handle *mycobacteria* in class II or III BSC to avoid their associated possible LAI [34]. Recently, Wurtz et al. [36] surveyed laboratory-acquired infections around the world in BSL-3 and BSL-4 laboratories. Out of 23 laboratories, only four reported around fifteen LAIs cases caused by four different pathogenic cultures. These have been classified as BSL-3 bacteria and belong to the species (1) *Mycobacterium tuberculosis* (ten cases), (2) *Coxiella burnetii* (two cases), and (3) *Brucella melitensis* (two cases), while other reported cased were caused by a BSL-2 virus. The percent distribution of the majority of the LAIs (73%) that occurred in a BSL-3 laboratory was as follows: microbiology activities (42%), followed by microscopy (22%), and cell culture (22%) (Figure 3) [36]. Also, the laboratory personnel should undergo an annual Mantoux purified protein derivative skin test or an interferon-γ release assay to demonstrate conversion. The persons with positive test results should be further investigated for active tuberculosis by chest radiography [37].

### 4.3. Other Bacterial-Associated LAIs and Prevention Strategies

In addition to Brucellosis mentioned above and *M. tuberculosis*, several other bacterial strains have also been reported to cause LAIs though with lower frequencies. Amid these bacterial agents, *Francisella tularensis* is a zoonotic infection and usually occurs not only as a glandular ulcer form but also as pneumonia. There are some reported cases of *F. tularensis* mediated LAIs in the literature, which are more frequently linked to the bacterial cultures rather than on infected animals or clinical material [34]. Considering the adverse consequences of antibiotic-based therapy treatment, appropriate vaccination along with accurate biosafety measures have been recommended as the most valuable tool to control these infections [38]. Microorganisms such as *Salmonella* or *Shigella* belonging to Enterobacteriaceae have also been recognized to cause LAIs [26]. Moreover, reports have shown that several other pathogenic bacterial agents like *Escherichia coli*, *Clostridium difficile*, or *Klebsiella* spp. may be classified as potential LAIs [39].

### 4.4. Viral-Associated LAIs and Prevention Strategies

In recent years, virus research is associated with widespread applications in biotechnological sectors, such as viral diseases, the development of novel vaccines, or GMOs. Despite scarce research investigation concerning virus associated LAIs, pathogenic infections with the human immunodeficiency hemorrhagic virus, West Nile Virus, Dengue, or Marburg virus have been reported in the literature [40,41,42]. Viral agents transmitted through blood and body fluids are responsible for most of the LAIs amongst healthcare employees in diagnostic laboratories [43]. Despite that, the viral hemorrhagic fevers provoke the greatest fear, these viruses are rare causes of laboratory infection [21,22]. Among the common blood-associated viruses, HBV is the leading cause of LAIs [43], and among all health care workers, the incidence of HBV infection in the United States is approximated to be 3.5–4.6 infections per 1000 workers [44]. Encouragingly, among the laboratory staffs, there were no reported cases of HBV infection in the two most recent inspections of LAIs in the UK [24,27]. These findings emphasized the implementation of universal precautions while handling blood specimens, developments in needleless devices, and the appropriate vaccination. During 2005–2006, there were 802 confirmed cases of HCV reported to the Centers for Disease Control and Prevention, with five occupational exposures to blood [45]. However, very few data were found on the occurrence of HCV among laboratory employees with only one case in the US and UK [24,25,27]. HIV infection related to contaminated blood or body fluids exposure consequences the paramount apprehension. From 1981 to 1992, HIV reports revealed a total of 32 healthcare workers in the US with occupationally acquired HIV infection. Among these, 25% of health care staffs were noted to be laboratory workers [46]. Therefore, correct biosecurity and biosafety procedures, immune control approaches, education and training, and specialized laboratory facilities should be adopted to reduce the potential risk of LAIs or viral-associated diseases [47].

### 4.5. Parasites Associated LAIs and Prevention Strategies

Parasite-associated LAIs are uncommon in the diagnostic microbiology laboratories [34,43]. Among the parasitic infections, LAIs caused by Leishmaniasis, fascioliasis, malaria, toxoplasmosis, trypanosomiasis, or schistosomiasis have been found to be the most adverse forms [48,49]. Nearly, 313 cases of LAIs, with a variety of blood and intestinal protozoa, have been reported [21,34,50]. Many of these cases occurred in reference and research laboratories. Among laboratory staffs and healthcare employees, a total of 52 malaria cases have been reported, with 34 cases reviewed by Herwaldt [50]. Out of these, 10, 9, and 15 cases were caused by *Plasmodium cynomolgi*, *P. vivax* and *P. falciparum*, respectively [21,50]. The direct contact or exposure to parasites in the laboratory presumably increases the potential risk for acquiring parasitic infections. Several causes including needlestick injuries, barehanded work in the open field are the common means associated with parasitic LAIs. Since parasitic diseases are commonly characterized by a prolonged asymptomatic period, laboratory employees working with parasites are advised to be tested intermittently [50]. Additionally, exceptional attention must be taken for childbearing women due to the hereditary transmission of some protozoan parasites [51].

### 4.6. Fungal-Associated LAIs and Prevention Strategies

The dimorphic fungi, *Blastomyces dermatitidis*, *Coccidioides immitis*, and *Histoplasma caspsulatum* are the primary causative agents for most of the fungal-associated LAIs in the US [21,22,43]. Although cutaneous infections are reported due to accidental inoculation, the majority of the LAIs occurred because of inhaling infectious conidia that led to pulmonary infection. The risk of fungal infection is probably lower in the mycology laboratories, because specimen handling is carried out in laminar-flow biological safety cabinets (BSCs), and culture plates are also sealed to avoid accidental opening. Nevertheless, infection risk is likely to increase on the aerobic culture bench, because *B. dermatitidis* and *C. immitis* colonies can grow on conventional culture media within 2–3 days. Therefore, clinicians suspecting dimorphic fungal-associated infections should immediately alert the microbiology laboratory [34].

## 5. Biological Risk Classification and Prevention Strategies

As mentioned above, there are numerous human health-related biological risks which are categorized based on their causative agents such as bacteria, fungi, viruses, parasites, or genetically modified organisms. Pathogenic microbes characterize only a fraction of hazardous wastes of high concern that originates during laboratory investigations. Whereas, the control implication is based on their probable risk to humans, animal, and agriculture. According to the literature, various types of hazardous wastes are directly or indirectly responsible for large-scale infections with enormous ecological and socio-economic consequences [52]. All laboratory employee working with pathogenic strains or biologically hazardous materials are prone to risk from clinical specimens and cultures. Therefore, it is essential to know the potential risk of the concern agents to the health of laboratory staff, human and animal population in case of an outbreak [53].

Based on their principal characteristics, route of disease transmission, and hazard to laboratory staff/employees and the community, the World Health Organization (WHO) developed a system to categorize microorganisms into four different groups [54] (Table 2). Biological agents of risk group 1 comprise microorganisms that are unlikely to cause any disease in man. Risk group 2 includes biological agents that can cause disease in humans and posture a serious menace for staffs but with negligible chances of dissemination among these workers or to the community. Prophylaxis and effective treatments are available against these diseases. Biological agents of risk group 3 represent serious disease/danger in humans and workers with the potential risk of dissemination among the community, but an effective treatment or prophylaxis is available. Finally, biological agents causing severe illness in humans and representing a serious menace to workers along with the probability of being spread to the community are included in risk group 4. For these, there is usually no effective treatment or prophylaxis available [54]. Considering the pathogenic effects to humans, and the potential hazard to the environment, the maximum containment measures and procedures should be subjected and followed to diminish all these risks [1]. Moreover, correct handling with specified containment facilities and equipment must be undertaken while dealing with infected [55].

## 6. Lessons Learned and Future Perspectives

Research is underway around the globe, both at national and international levels, to establish and implement biosafety and biosecurity procedures in all laboratory settings, such as clinical, microbiology, biotechnology, and bioengineering laboratories, to ensure the safety and reduce the risk of an outbreak. Though numerous biosafety principles and good laboratory practices manuals are available, their recommendations and procedures are essentially the similarities among these laboratories. International agencies such as WHO, FAO (Food and Agriculture Organization of the United Nations), OIE (World Organization for Animal Health), and other related associations published biosafety guidelines manuals in cooperation with professional groups to assist developing countries in the publication of their biosafety manuals. Based on literature evidence, three internationally recognized regulatory agencies, i.e., (1) WHO, (2) FAO and (3) OIE, have recommended well-organized and proper biosafety and biosecurity precautions, which could improve the standardization of criteria to tackle LAIs [56].

It is of profound importance that the protocols adopted by laboratory staff in manipulating genetic of microorganisms should be according to biosafety and biosecurity guidelines to circumvent potential propagation to the atmosphere. Proper identification of potential yet unknown hazards associated with the genetic manipulation techniques is also necessary. In addition to researchers’ responsibilities for delivering awareness of the risks and hazards, institutions should also provide the technical and human resources mandatory to guarantee all biosafety and biosecurity measures [57]. Specialized training should be conducted to address pathogenic microorganisms that are being manipulated and studied. Besides, an appropriate immune control policy should be employed for the entire laboratory staff [58]. Risk group 4 pathogens such as certain arenaviruses and Hendra viruses often cause lethal diseases in infected humans. Pathogens currently handled at biosafety level for risk group 3 and 4 pose thoughtful risks to laboratory staff. Therefore, these laboratories must imitate the most rigorous safety procedures [47].

In the near future, the development of new drug formulations and vaccines for the treatments of emerging and re-emerging diseases will involve the rapidly growing innovative advances in biotechnology and nanotechnology. Specific education and training programs for laboratory workers together with the design and construction of proper laboratory facilities must be followed to evade LAI [59]. The researches and the scientific community should learn from past lessons to anticipate future complications associated with biological risks and LAIs. Another important lesson which we must learn from is non-reporting LAIs practice in the past. It could pose a serious, life-threatening risk of disease transmission and/or spread over from infected laboratory or staff to communities and the environment. In this context, all types of laboratories discussed in earlier sections should encourage their workers to report LAIs cases, with utmost care, from all safety levels. This practice will provide useful information to the governing bodies and regulatory authorities to monitor and tackle the unintended release of threatening pathogens, around the globe.

## 7. Concluding Remarks and Future Perspectives

In conclusion, LAIs represent a serious occupational risk to laboratory staffs/workers, particularly those working in the clinical/microbiology laboratories. Exposures may occur unintentionally or following accidental inoculation, but every exposure does not result in infection. The diagnostic and clinical laboratories are on the front line for the identification of outbreaks of emerging infectious diseases. Therefore, these laboratories require proper biosafety and biosecurity preventive strategies to protect staff health and from pathogenic infection. There is a dire need to implement biosafety/biosecurity culture in all types of clinical laboratories at large and microbiology, mycology, bacteriology, and virology-oriented laboratories, in particular, rather than strengthening the regulatory perspectives alone. The biosafety program includes proper staff education, training, and awareness to guarantee proper apprehension and execution of biosafety procedures to ensure the maintenance of a safe working environment for the laboratory staff and the wider community. Moreover, each laboratory should plan and implement its pathogen-specific preventative measures to upgrade biosafety/biosecurity in reducing the probabilities of LAI incidences. A risk-based strategy should be applied to all biosafety programs focusing on pathogen-based factors (i.e., routes of infection, infectious dose) while working with pathogenic organisms. Finally, health testing and vaccinations, and post-vaccination adverse effects and symptom checking on a routine basis are suggested.

## Figures and Tables

**Table 1:** Summary of the LAI reports in the Asia-Pacific region.

Year	Country	Microorganism	Affected Worker	Laboratory Type/Level
2016	Taiwan	*Ralstonia pickettii*	-	-
2014	South Korea	Dengue	Laboratory staff	Research/BSL2
2011	Australia	Dengue	Scientist	Research/BSL2
2010	India	Buffalopox virus (BPXV) (Z)	Researcher	-
2009	Malaysia	*Brucella melitensis*	Laboratory staff	Clinical
2006	Taiwan	*Shigella* spp. (Z)	Graduate student	Research
2006	PR China	Seoul virus and hantavirus (Z)	8 postgraduate students	Research
2004	Taiwan	Dengue type 1	Graduate student	Research
2004	Taiwan	SARS-CoV (Z)	Researcher	Research
2004	PR China	SARS-CoV (Z)	8 human cases, 1 died	Research
2003	Singapore	SARS-CoV (Z)	Graduate student	Research/BSL3
2002	Taiwan	*Arthroderma benhamiae* (Z)	Scientist	Research
2002	Australia	*S. aureus*, MRSA, EMRSA (Z)	Laboratory staff	Clinical
2001	Japan	*Arthroderma benhamiae* (Z)	Researcher	Research
2000	South Korea	*Orientia tsutsugamushi* (Z)	Worker	-
1999	Taiwan	*Vibrio parahaemolyticus* (Z)	Laboratory staff	-
1998	Japan	*Helicobacter pylori* (Z)	Bacteriologist	-
1996–2000	Australia	*Brucella suis* (Z)	Various	Clinical
1996	Malaysia	*Salmonella typhi*	Laboratory staff	-
1992	Australia	*Pseudomonas pseudomallei* (Z)	3 Laboratory staff	Diagnostic
1990	South Korea	*Rickettsia typhi* (Z)	Laboratory staff	Clinical
1990	India	*Mycobacterium leprae* (Z)	Worker	Clinical
1989	South Korea	*Rickettsia typhi* (Z)	Laboratory staff	Research
1987	Australia	Newcastle disease virus (Z)	Laboratory staff	Research/BSL3
1986	Australia	*Brucella melitensis* (Z)	Researcher	Research
1985	Japan	*Mycobacterium tuberculosis* (Z)	Pathologist	Research
1982	Australia	*Shigella flexneri* (Z)	Laboratory staff	Clinical

Note: Data collected from the American Biological Safety Association [18].

**Table 2:** WHO and NIH risk group classifications. Reproduced from WHO [54].

Risk Group	Individual Risk	Community Risk	Description
1	Low	Low	A microorganism that is unlikely to cause human or animal disease.
2	Moderate	Low	A pathogen that can cause human or animal disease but is unlikely to be a serious hazard to laboratory workers, the community, livestock or the environment. Laboratory exposure may cause serious infection, but effective treatment and preventive measures are available, and the risk of spread of infection is limited.
3	High	Low/moderate	A pathogen that usually causes serious human or animal disease but does not ordinarily spread from one infected individual to another. Effective treatment and preventive measures are available.
4	High	High	A pathogen that usually causes serious human or animal disease and can be readily transmitted from one individual to another, directly or indirectly. Effective treatment and preventive measures are not usually available.
